# Retraction: Infectious Offspring: How Birds Acquire and Transmit an Avian Polyomavirus in the Wild

**DOI:** 10.1371/annotation/255a6a86-fee6-4663-a23f-36e392e87656

**Published:** 2013-05-13

**Authors:** Jaime Potti, Guillermo Blanco, Jesús Á. Lemus, David Canal

The authors Drs Potti, Blanco and Canal retract this publication. 

The Ethics Committee of the Spanish Superior Council of Scientific Research (CSIC) has carried out a formal investigation in relation to concerns about potential scientific misconduct by Jesús A. Lemus, the third author of this article. The investigation has questioned the validity of the laboratory analyses conducted by Dr. Lemus. Although the published field and statistical procedures reported in our article are correct, we have been unable to clarify in which external laboratories Dr Lemus conducted the molecular analyses and which primers he used.

We have now partially replicated the work by submitting blood aliquots of the same samples employed in the original study to two different independent laboratories to assess the validity of the published results on avian polyomavirus (APV) in pied flycatchers. In both cases, replicate samples with different IDs were also submitted as controls.

The first re-analysis (Technoscience, Seville, Spain) used the protocol followed by Tomasek et al. [1] on 40 samples previously scored by Dr Lemus as 27 positive and 13 negative for APV. Only 37% (10 out of 27) of the samples originally scored as positive for APV were also positive in the re-analysis carried out by Technoscience, while the consistency was greater for negative scores (86.5%, 11 out of 13 samples scored negative for APV in the re-analysis). 

A second, more extended re-analysis (NBT, Seville, Spain) used the protocol by Johne et al. [2] on 276 of the original samples. In addition, the NBT laboratory ran additional PCRs using their own designed primers for a conserved region of APV. All of these analyses have returned negative scores for APV presence in all samples.

In the light of the lack of consistency in relation to the prevalence of APV between the published results and the re-analyses undertaken, the authors Drs Potti, Blanco and Canal wish to retract this article. We sincerely apologize for any inconvenience to the readership of PLOS ONE.

References

1. Tomasek O, Kubicek O, Tukac V (2008) Comparison of three template preparation methods for routine detection of beak and feather disease virus and avian polyomavirus with single and nested polymerase chain reaction in clinical specimens. Avian Pathol. 37: 145-149. doi:10.1080/03079450801902047 

2. Johne R, Enderlein D, Nieper H, Müller H (2005) Novel polyomavirus detected in the feces of a chimpanzee by nested broad-spectrum PCR. J. Virol. 79:3883-3887. doi:10.1128/JVI.79.6.3883-3887.

